# Powder Reuse in Laser-Based Powder Bed Fusion of Ti6Al4V—Changes in Mechanical Properties during a Powder Top-Up Regime

**DOI:** 10.3390/ma15062238

**Published:** 2022-03-17

**Authors:** Ryan Harkin, Hao Wu, Sagar Nikam, Shuo Yin, Rocco Lupoi, Wilson McKay, Patrick Walls, Justin Quinn, Shaun McFadden

**Affiliations:** 1 School of Computing, Engineering and Intelligent Systems, Ulster University, Derry BT48 7JL, UK; r.harkin1@ulster.ac.uk (R.H.); s.nikam@ulster.ac.uk (S.N.); jp.quinn@ulster.ac.uk (J.Q.); 2 Department of Mechanical, Manufacturing and Biomedical Engineering, Trinity College Dublin, D02 PN40 Dublin, Ireland; yins@tcd.ie (S.Y.); lupoir@tcd.ie (R.L.); 3 Laser Prototypes Europe Ltd., Belfast BT5 6QR, UK; wilson@laserproto.com (W.M.); patrick@laserproto.com (P.W.)

**Keywords:** laser-based powder bed fusion, ELI (Grade 23) Ti6Al4V, powder recycling, mechanical properties, Tabor’s relationship

## Abstract

The properties of Extra Low Interstitials (ELI) Ti6Al4V components fabricated via the laser-based powder bed fusion (L-PBF) process are prone to variation, particularly throughout a powder reuse regime. Interstitial pick-up of interstitial elements within the build chamber during processing can occur, most notably, oxygen, nitrogen, and hydrogen, which can impair the mechanical properties of the built component. This study analyses ELI Ti6Al4V components manufactured by the L-PBF process when subjected to a nine-stage powder reuse sequence. Mechanical properties are reported via hardness measurement and tensile testing. Results showed that from 0.099 wt.% to 0.126 wt.% oxygen content, the mean hardness and tensile strength increased from 367.8 HV to 381.9 HV and from 947.6 Mpa to 1030.7 Mpa, respectively, whereas the ductility (area reduction) reduced from around 10% to 3%. Statistical analysis based on the empirical model from Tabor was performed to determine the strength–hardness relationship. Results revealed a significant direct relationship between tensile strength and Vickers hardness with a proportionality constant of 2.61 (R-square of 0.996 and *p*-value of 6.57 × 10^−6^).

## 1. Introduction

**Figure 1:**
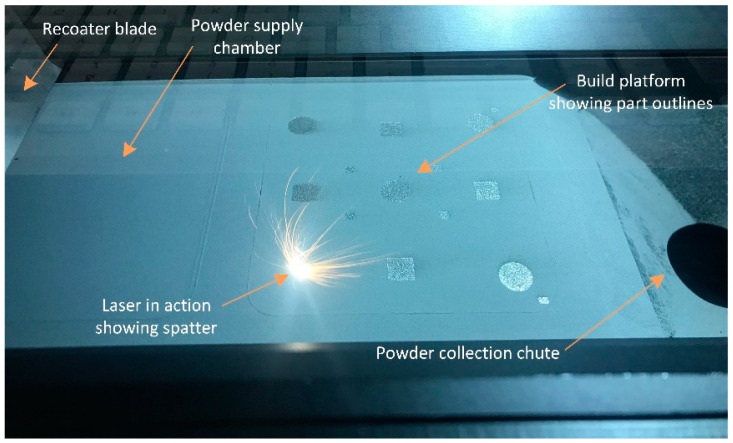
The laser-based powder bed fusion (L-PBF) apparatus and process.

Laser-based powder bed fusion (L-PBF) is a subcategory of additive manufacturing processes that uses a focused laser beam to selectively melt consecutive layers of metallic powders to produce three-dimensional components. Figure 1 shows an image of the L-PBF process used in this study. At the start of each layer, a recoater blade spreads a thin layer of powder from the powder supply chamber to cover the build platform. Any excess powder for the recoating process is collected in the chute. The recoater blade returns to its staring position, and the laser scans across the build platform (as shown in Figure 1) to selectively consolidate the powder onto the existing solid substrate beneath the powder layer. The supply chamber and the build platform are both piston-operated; hence, the supply chamber is elevated by a fixed amount to present fresh powder for recoating. The build platform descends by a fixed amount to accept a new coating of powder. The process is repeated until the part is fully built.

As a result of the direct melting–consolidation mechanism, highly complex geometries can be manufactured [1,2]. However, the process is complex, and many processing parameters can be selected. It has been stated that there are up to 157 processing parameters associated with the L-PBF process, which can be categorized, accordingly, as preprocessing, in situ, and postprocess parameters [3].

A significant preprocess factor to consider is the condition of the feedstock powder, typically determined by performing characterization tests classified in relation to the powder morphology, chemistry, and microstructure [4]. Due to the challenging economics and to improve the sustainability of the L-PBF process, powder is routinely reused within a top-up regime [5,6] after sieving and blending with virgin powder.

### 1.1. Additively Manufactured Titanium Alloys

Parts can be produced using Ti6Al4V alloy powder. It is particularly important to characterize titanium alloy powder during the reuse regime, as there is a greater opportunity for powder property variation to occur. Molten Ti6Al4V has a high affinity to elements that form interstitial solid solution, mainly oxygen, nitrogen, and hydrogen [5]. Most notably, interstitial pick-up occurs during the formation of a melt pool, as this is when titanium is at its most reactive state [7]. The interstitial elemental levels within the titanium components can significantly increase across a powder reuse regime to above the maximum acceptable limit of Grade 23 [5]. Furthermore, it has been demonstrated that the content of the interstitial oxygen within powder captured from the sieving process can be over three times the Grade 23 threshold [6]. Velasco-Castro et al. [8] reported a significant increase in oxygen content within Ti6Al4V lattice structures, with an increasing number of laser beam passes providing a comparison between lattice structures fabricated with one and nine laser beam passes, resulting in the oxygen increased from 0.412 wt.% to 1.15 wt.%.

The mechanical properties of titanium alloy components largely depend on their microstructure, defect levels, and chemical composition [9]. Ti6Al4V is an α + β titanium alloy, and due to the rapid cooling rate (over 410 °C/s) that occurs during the L-PBF process, its as-built components typically exhibit an acicular α’ martensitic microstructure [10,11]. This form of microstructure has been shown to be associated with high strength but low ductility [12]. As oxygen and nitrogen are interstitial elements, the pick-up of such elements during processing will lead to an increase in the c/a ratio and, in turn, an increase in tensile strength and hardness but a decrease in ductility [13,14]. Oh et al. [14] reported an increase of oxygen content within Ti6Al4V components from 1170 ppm to 3360 ppm, resulting in a 20% increase in the hardness value. They suggested that the increase of strength was mainly contributed by the interstitial solid solution of oxygen, but the details of the heat treatments were not included. In contrast, Rousseau et al. [15] reported that a higher oxygen level could promote the formation of grain boundary Ti-α and lead to the refinement of Ti-α laths. This resulted in the increase of the tensile strength without affecting the elongation. Carroll et al. [16] showed that the yield strength and tensile strength of Ti6Al4V components increased by 16 Mpa and 9 Mpa, respectively, whenever the oxygen content increased by 0.0124 wt.%. They mentioned that the interstitial oxygen element strengthened and stabilized the Ti-α phase.

### 1.2. Relationships between Mechanical Properties

Research has focused on investigating the relationship between Vickers hardness and tensile properties [17,18,19]. An early study by Tabor suggested that for nonstrain-hardening metals, the yield stress in Mpa, *σ*, is directly related to the Vickers hardness, *H*V [17]:

*σ* ≈ *K·H*V, (1)

where *K* is a constant of proportionality generally thought to be around 3 or 3.27 but must be established empirically. Later studies have expanded on this relationship to give the relationships between tensile strength and hardness of Ti6Al4V. Hickey, Jr. [18] suggested a relationship between tensile strength and Vickers hardness of annealed Ti6Al4V by performing the linear least square fitting with a constant to experimentally determined data. Recently, Keist and Palmer [19] modified this relationship and developed a tensile strength–Vickers hardness relationship of Ti6Al4V fabricated by directed energy deposition after heat treatment. These simple relationships are useful, as they give a practical way to estimate tensile properties from hardness data.

### 1.3. Aims and Objectives

The main aim of this study is to evaluate the effects of elevated interstitial elemental concentrations (measured within the powder during a top-up reuse regime previously presented by the authors [5]) on mechanical properties of L-PBF components. A second aim is to utilize the empirical strength–hardness relationship to investigate the relationship (Equation (1) or similar) between the tensile strength and hardness of the L-PBF components. The specific objectives include:

1. To evaluate the effect of increasing interstitial elements in the Ti6Al4V powder on the Vickers hardness of the selected built components.
2. To analyze the impact of interstitial elements within the reused powder on the tensile properties.
3. To establish the strength–hardness relationship by adapting the existing empirical model and to compare the model from other literature.

This manuscript will perform regression analysis to determine the relationship between the tensile strength and hardness based on the model suggested by Tabor and with other relationships found in literature. Discussions will be made to investigate the nature of strengthening as a result of interstitial element pick-up and to compare the new results to other studies.

## 2. Materials and Methods

### 2.1. Feedstock Powder

**Figure 2:**
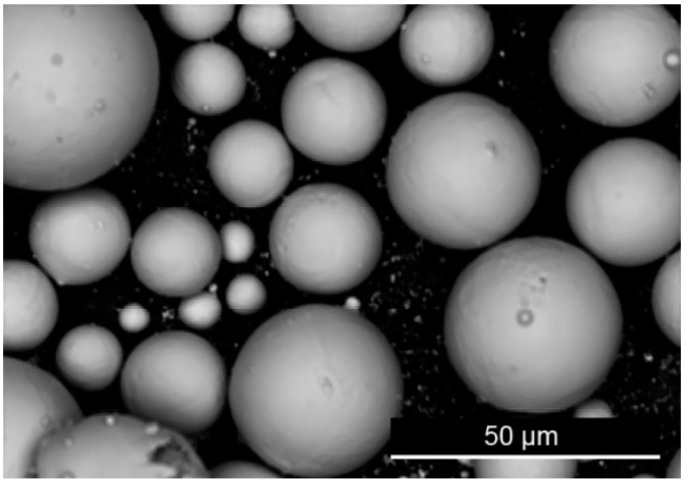
Scanning electron microscope image with magnification at ×1000.

Plasma-atomized Extra low Interstitials (ELI, Grade 23, Carpenter Additive, Widnes, UK) Ti6Al4V powder was used throughout this study. Figure 2 shows an image of the supplied powder that is representative of the spherical morphology. The powder was subjected to 9 reuse cycles within a top-up regime as reported elsewhere [5]. All details relating to the sieving and top-up quantities are presented in [5]. Chemical compositional analysis was carried out by inert gas fusion along the guidelines of ASTM E1409-13 for oxygen and nitrogen [20] and ASTM E1447-09 for hydrogen [21]. Inert gas fusion services (Carpenter Additive, Widnes, UK) were supplied by a commercial laboratory that were required to comply with ISO 17025 [22]. The oxygen, nitrogen, and hydrogen content measured prior to each cycle is provided in Table 1. The Grade 23 threshold for oxygen is 0.13 wt.%, which was recorded at cycle 8. Regression analysis was performed on the oxygen content versus the reuse stage, which gave a *p*-value of 7.9 × 10^−5^ on the slope; hence, since *p* < 0.05, we can say that this was a significant increasing trend. Oxygen content should be considered as having exceeded the Grade 23 maximum requirement by the end of the top-up regime. The nitrogen levels showed an increasing trend with a *p*-value of 7.6 × 10^−5^ on the slope. However, nitrogen was maintained well below the threshold value for Grade 23, which is 0.05 wt.% (highest recorded nitrogen level was 0.02 wt.%). Hydrogen showed no discernable trend and was maintained well below the maximum Grade 23 requirement of 0.012% throughout (at 0.0024 wt.%, the highest recorded hydrogen level was an order of magnitude lower than the threshold).

Several other properties of the powder were reported in [5], such as particle size distribution through laser size diffraction, morphology through microscopy (electron and optical) and quantitative shape analysis, density (tap and apparent), and flowability through Hall flow rate testing. All of these parameters remained relatively steady throughout the top-up regime.

On the basis of the data presented in [5], the main parameter that was observed to change significantly and which exceeded its maximum threshold value was oxygen content. Hence, the following analysis concentrates, primarily, on the effect of elevated oxygen on the mechanical properties of the manufactured components.

### 2.2. Laser-Based Powder Bed Fusion (L-PBF) Parameters

Fabrication of the L-PBF components was performed using an Mlab Cusing R (GE Additive, Lichtenfels, Germany) machine with an 80 mm × 80 mm × 90 mm build chamber (as shown in Figure 1). The chamber was continuously purged with argon, which controlled the oxygen levels during fabrication to be approximately below 0.1 wt.%. An alternating 45° between-layers scanning strategy was selected. The process parameters that remained constant throughout were laser power, 95 W; scanning speed, 900 mm/s; hatch spacing, 100 µm; and a layer thickness, 25 µm.

### 2.3. L-PBF Build Layout

Two fabrication designs were used within the previous study [5], known as pillar build (build cycles 1, 4, 6, and 8) and test-part build (build cycles 2, 3, 5, 7, and 9). Only the test-part builds were used for testing the effects of oxygen content on the mechanical properties; hence, this study reports on build cycles 2, 3, 5, 7, and 9 only for mechanical data. It is assumed that all intermediate data can be interpolated using the information provided.

**Figure 3:**
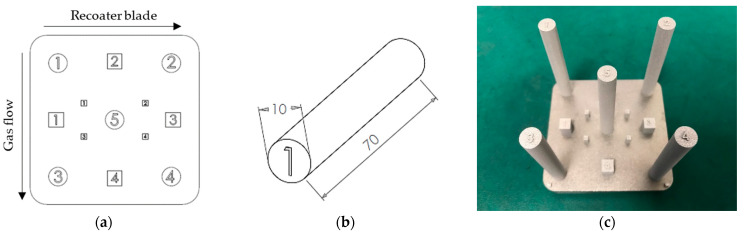
Test-part build design providing (**a**) top view of the test-part build, (**b**) the geometry of a cylindrical samples used for tensile and hardness testing, and (**c**) final parts on the build plate.

Figure 3 provides the detail on the geometry of the test-part build design. The part design included a variety of characterization components of different heights that were numbered to give a position on the build substrate with respect to the recoating blade and gas flow direction. Standing at 70 mm, the cylindrical parts were much taller than the other cubic elements (5 mm and 15 mm tall, respectively).

### 2.4. Postprocessing

As-built components on the build platform (GE Additive, Lichtenfels, Germany) were subjected to a stress-relieving heat treatment according to ASTM F3001 Class F, that is, as agreed with the manufacturer. The stress relief consisted of a three-stage heat treatment cycle below the beta transus temperature, whereby an argon-filled furnace was heated from room temperature to 694 °C, with a ramp up factor of 250 °C/h, and was maintained for 2 h and 42 m to be held in the range of 694 °C to 704 °C. Afterwards, the components were furnace-cooled to room temperature.

After heat treatment, electrical discharge machining (EDM) was performed to separate the parts from the platform. The cutting parameters used were feed rate, 2.8 mm/min; voltage, 60 V; and current, 2 A. An allowance of 2.5 mm additional length was added to all build geometries to account for the EDM Kerf width.

### 2.5. Hardness Testing and Etching

A Future-tech FM-800 Micro hardness tester (Future-tech, Kawasaki, Tokyo, Japan) was used, and a load of 1 kg and a dwell time of 10 s were applied. Cylinder 5 (shown in Figure 3a) from builds 2, 3, 5, 7, and 9 from each test-part build were tested. Initially, the parts were cut down along the vertical cross-sections and hot-mounted. The samples were ground in stages (water-assisted) using SiC papers from P240 up to P2500, then polished using 3 µm diamond suspension and finished with 0.06 µm colloidal silica suspension. The samples were tested along the centerline along the vertical direction relative to the substrate. The distances between each indentation measurement were kept to 1 mm, and, on average, a total number of 65 indents per sample were measured. A region of a sample was etched using Kroll’s reagent to confirm the microstructure present.

### 2.6. Tensile Testing

Four cylindrical components (cylinders 1 to 4 in Figure 3a) from build cycles 2, 3, 5, 7, and 9 were machined down to produce dog bone-shaped tensile bars, in accordance with the ASTM E8/m standard. An Instron universal materials tester (Instron, Norwood, MA, USA) was used to carry out tensile tests with a crosshead speed of 1 mm/min selected.

## 3. Results

### 3.1. Microstructure

**Figure 4:**
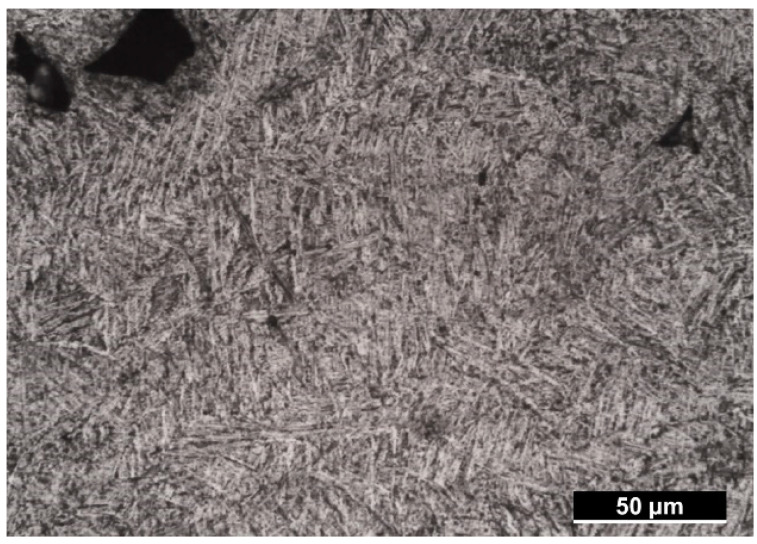
Representative microstructure after stress relief heat treatment. Basket weave microstructure is apparent.

Figure 4 shows the microstructure that was observed after stress relief heat treatment. The microstructure was representative of basket weave α + β. The images show laths of α and β phases.

### 3.2. Hardness

**Figure 5:**
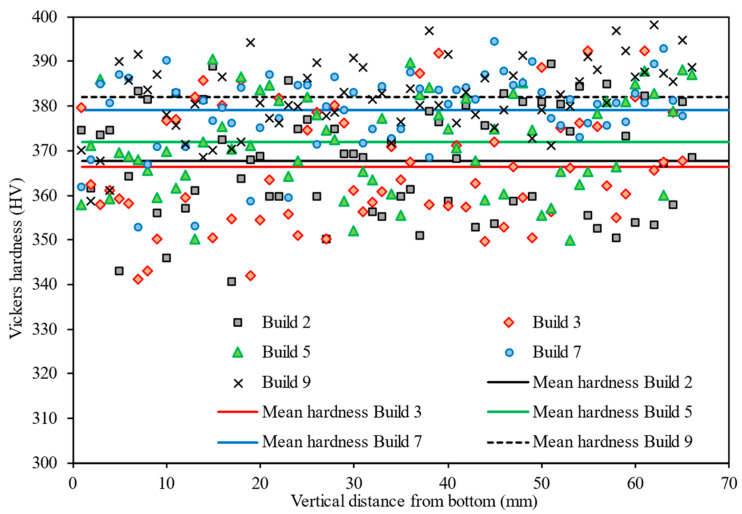
Vickers hardness measured as a function of height from the substrate for build cycles 2, 3, 5, 7, and 9. The solid lines represent the mean hardness of each component.

Figure 5 reports the hardness values across the build height of the cylindrical bars (cylinder 5) from selected builds. The findings showed that the hardness remained relatively constant along the axial direction with variance as shown.

**Figure 6:**
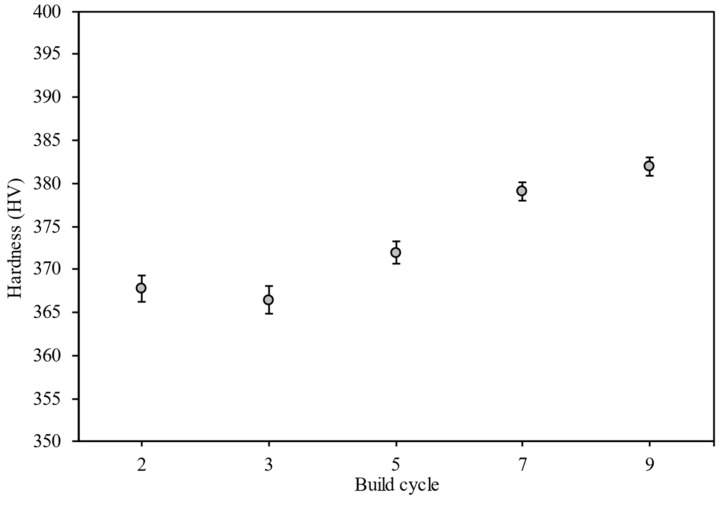
Mean Vickers hardness measured along the vertical centerline of each central cylinder. Error bars show standard errors.

The mean hardness values measured at each available cycle stage are presented in Figure 6 with standard error bars shown (standard error of mean).

**Figure 7:**
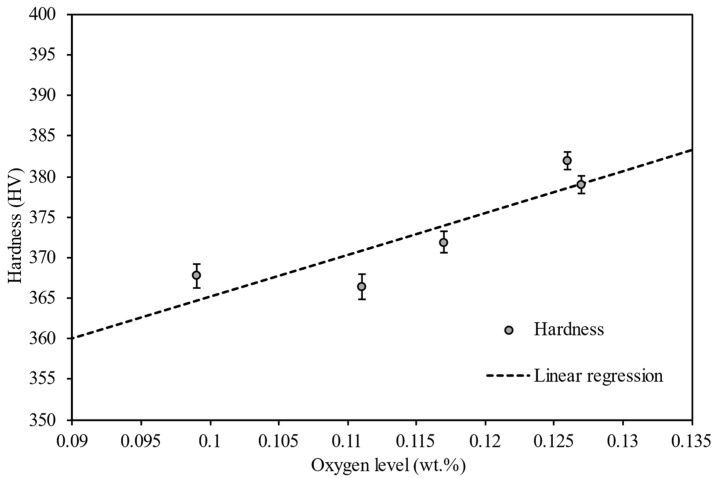
Mean Vickers hardness against oxygen content within recycled Ti6Al4V powder.

The mean Vickers hardness against oxygen content within reused powder is presented in Figure 7 along with a line of best fit from the regression analysis.

### 3.3. Tensile Strength

**Figure 8:**
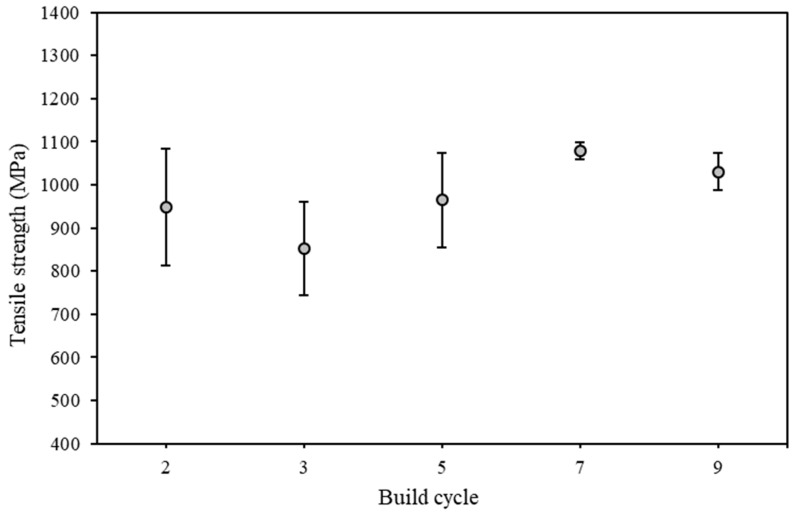
Mean tensile strength measured for build cycles 2, 3, 5, 7, and 9 with standard error bars.

Figure 8 shows the mean tensile strength at the available cycle stages with the standard error.

**Figure 9:**
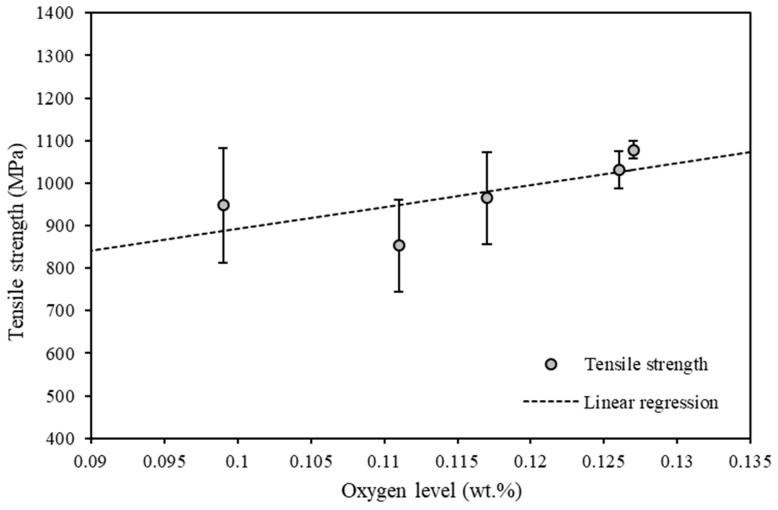
Tensile strength versus oxygen content within reused Ti6Al4V powder.

The mean tensile strength against oxygen content within the recycled powder is presented in Figure 9 along with the corresponding line of best fit.

**Figure 10:**
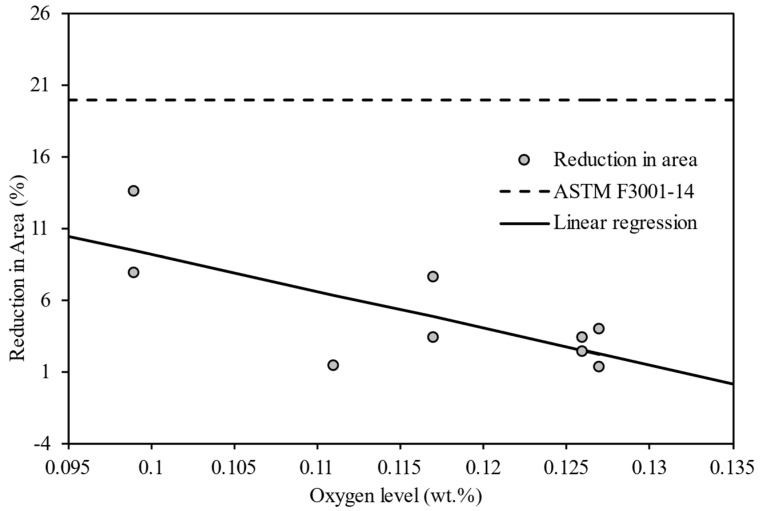
Reduction in area of the tensile tested samples against oxygen level of Ti6Al4V powder. The dashed line shows the value provided by ASTM F3001-14 [23]; the solid line indicates the linear regression result.

Figure 10 displays the relationship between the reduction in area of the tensile tested samples and the oxygen level. It shows an apparent decreasing trend of the reduction in area with an increasing level of oxygen, and all measured values were recorded to be well below the standard value (20%) provided by ASTM F3001-14 for ELI Ti6Al4V components receiving the stress-relieving treatment (Class F heat treatment) [23].

Table 2 summarizes the mean hardness and tensile strength for selected build cycles of 2, 3, 5, 7, and 9. The value after the plus–minus symbol indicates the standard error.

The details of the regression analyses are provided in Table 3. The linear regression parameters (intercept and slope) for hardness, tensile strength, and ductility versus oxygen content are presented along with the R-square and *p*-value for the slope of the fitting line.

**Figure 11:**
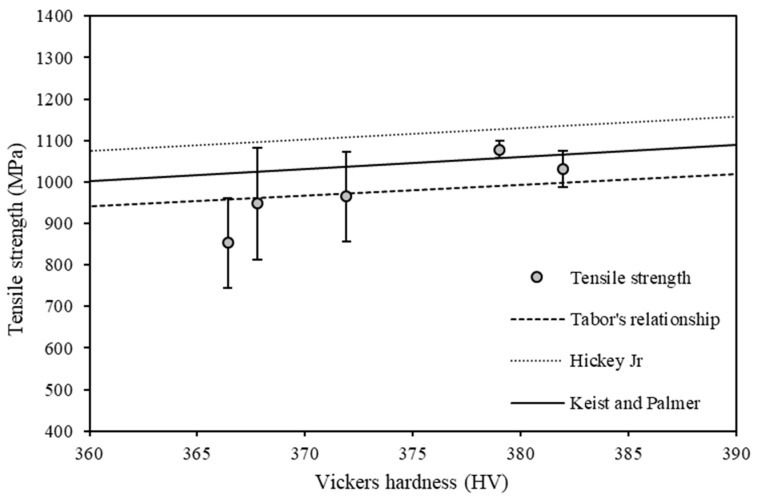
Mean tensile strength plotted against the mean Vickers hardness. Straight lines show the results of linear regression analysis.

Figure 11 displays mean Vickers hardness against the mean tensile strength. To establish the empirical model of Tabor (similar to Equation (1)) [17], linear regression analysis with a zero intercept was performed to determine the direct relationship between yield strength and hardness. The linear relationships suggested by Hickey, Jr. [18] and Keist and Palmer [19] are also included in the graph for comparison purposes.

Table 4 provides a summary of the mechanical property relationships (hardness versus tensile strength) for the various fitted data. Tabor’s relationship was determined through the current study; hence, the R-square and *p*-value are presented. Hickey, Jr., and Keist and Palmer data are reported from literature.

## 4. Discussion

Linear regression analyses were performed to quantify the effect that the oxygen content within the powder had on the hardness and tensile strength of Ti6Al4V components manufactured within the L-PBF process. The results obtained from the linear regression analyses are provided in Table 3. For the hardness versus the oxygen level, there is an apparent upward linear trend with a sound R-square value of 0.76, but the p-value of 0.054 demonstrates significance (after rounding to two decimal places, given *p* = 0.05). The *p*-value of 0.194 for tensile strength against oxygen level was found to be above the level required to prove statistical significance. The tensile test data suffered from large variances over a maximum of four tests per platform. In some cases, the tensile test failed outside of the gauge marks, but at no stage did we consider less than three tensile tests at any reuse stage. Hence, the data for tensile testing suffered from low sample size, and this made significance difficult to confirm. Nevertheless, the *p*-value of 0.023 for the reduction in area against the oxygen level further did confirm a significant reduction in ductility as the oxygen level increased.

Oh et al. [14] showed that the presence of interstitial oxygen in solid solution modified the lattice parameters or c/a ratio. Any change in the lattice parameter affects the ability of dislocations to glide, therefore causing an increase in the strength but a reduction in ductility. The strengthening of Ti6Al4V is linked to the elevation of oxygen in an interstitial solid solution. However, the decrease in ductility reported here seems to be more pronounced than in other studies [24,25]. However, it should be noted that our work reports reduction in area, whereas the work of others reports on percent elongation. In ASTM F3001, an elongation of 8% is equivalent to a reduction of area on 20%. Hence, given that both measures share the zero datum for no ductility, the reduction in area is apparently more sensitive to changes than elongation. Hence, our results may seem more pronounced for this reason. We also ignore the effect of increased nitrogen, which may have an additional interstitial strengthening effect in combination with the elevated oxygen levels.

The reported Vickers hardness of annealed or additively manufactured Ti6Al4V after HIP heat treatment ranges between 320 HV and 380 HV [10,14,19]. However, the highest hardness found in the present work approached 400 HV, which was higher than this range. This was because the samples in this study were only subject to a stress-relieving heat treatment in a low-temperature range (up to 704 °C). It has been demonstrated elsewhere that this stress-relieving heat treatment is too low to alter the α’ brittle martensite structure of the as-built state, which results in higher hardness levels of the manufactured component (408 HV) [10].

To determine the statistical significance of the relationship between tensile strength and hardness, a regression analysis with zero intercept—based on the empirical model from Tabor—was performed [17]. Table 4 shows the results of the analysis in Figure 11 and the regression analysis with nonzero intercepts reported in [18,19]. The linear regression analysis established a satisfactory linear relationship (R-square of 0.996) and a statistically significant relationship (*p*-value of 6.57 × 10^−6^). The slope obtained by our analysis was smaller than the other two studies, but the regression line shows the best fit, among others, as indicated by Figure 11. The analysis in this study has proved the feasibility of the Tabor’s relationship; however, it has been shown that the model should be modified on a case-by-case basis, with the relationship of Keist and Palmer also showing good agreement.

ASTM F3001-14 states that Ti6Al4V (Class F) parts should meet the requirements for mechanical properties, which are tensile strength of 825 MPa and area reduction of 20%. The parts in this analysis managed to exceed the requirement for tensile strength but did not meet this minimum requirement on area reduction because of defects in the finished parts. Lack of Fusion (LoF) defects were found to exist consistently throughout all build cycles at a volume fraction of approximately 4%. These LoF defects were due to the hatch spacing being too large for the given laser power and scan speed. Nevertheless, it is argued here that the hardness and ductility results do show significant trends that relate solely to the elevated oxygen levels present in the reused powder even as the defect level remained unchanged throughout. Furthermore, three subsequent build cycles were performed where the hatch spacing was reduced, and LoF was significantly reduced. In this case, the minimum requirements for strength and ductility in ASTM F3001-14 were approached with a reduction in area of 19.52% and tensile strength of 1167 MPa. The outcome of this research is beyond the scope of the current manuscript and is under preparation for publication elsewhere.

## 5. Conclusions

The main aim of this study was to evaluate the effects of elevated interstitial elemental concentrations (measured within the powder during a top-up reuse regime previously presented by the authors [5]) on mechanical properties of L-PBF components. Components were fabricated and tested at selected cycles from a total number of nine top-up reuse cycles. The powder characterization showed that only oxygen and nitrogen levels showed a changing trend, but only oxygen exceeded the threshold limit for Grade 23 titanium alloy. Hence, the study focused on the effect that elevated oxygen in the powder had on the hardness and tensile properties. It was shown that, primarily, the oxygen content did change the mechanical properties in line with the expectations of an interstitial solid solution strengthening mechanism. It is suggested that nitrogen may have also played a secondary role in the strengthening mechanism, and this should be investigated further.

A second aim was to utilize the empirical strength–hardness relationship to investigate the relationship between the tensile strength and hardness. Results revealed a direct ratio relationship between tensile strength and Vickers hardness. The analysis further proved the feasibility of using Tabor’s relationship to estimate tensile strength using Vickers hardness measurements. However, it was shown that the regression model of Keist and Palmer also shows good agreement.

## Figures and Tables

**Table 1:** Average chemical composition of the interstitial elements at each build cycle throughout a previously studied top-up reuse regime [5].

Build Cycle	1	2	3	4	5	6	7	8	9
O (wt.%)	0.095	0.099	0.111	0.114	0.117	0.123	0.127	0.130	0.126
N (wt.%)	0.014	0.015	0.0159	0.0173	0.0175	0.02	0.02	0.02	0.019
H (wt.%)	0.0020	0.0018	0.0023	0.0024	0.0023	0.0002	0.0002	0.0001	0.0004

**Table 2:** Mean Vickers hardness and tensile strength at each specified build cycle.

Build Cycle	2	3	5	7	9
Vickers hardness (HV1)	367.8 ± 1.5	366.4 ± 1.6	371.9 ± 1.3	379.0 ± 1.1	381.9 ± 1.0
Tensile strength (MPa)	947.6 ± 135.4	852.8 ± 108.7	964.6 ± 109.1	1078.4 ± 20.1	1030.7 ± 44.0

**Table 3:** Regression analysis of the mean hardness vs. oxygen level and tensile strength vs. oxygen level, respectively.

Relationship	Intercept	Slope	R-Square	*p*-Value
Hardness vs. oxygen level	313.6	515.4	0.760	0.054
Tensile strength vs. oxygen level	377	5150	0.481	0.194
Reduction in area vs. oxygen level	35.0	−258.1	0.55	0.023

**Table 4:** Results of the regression analysis based on Tabor’s relationship and from [18,19].

Analysis	Intercept	Slope	R-Square	*p*-Value
Tabor’s relationship	-	2.61	0.996	6.57 × 10^−6^
Hickey, Jr.	96.5	2.72	-	-
Keist and Palmer	−56	2.94	-	-

## Data Availability

All the data is available within the manuscript.
